# Heme activates TLR4-mediated inflammatory injury via MyD88/TRIF signaling pathway in intracerebral hemorrhage

**DOI:** 10.1186/1742-2094-9-46

**Published:** 2012-03-06

**Authors:** Sen Lin, Qing Yin, Qi Zhong, Feng-Lin Lv, Yu Zhou, Jing-Qi Li, Jing-Zhou Wang, Bing-yin Su, Qing-Wu Yang

**Affiliations:** 1 Department of Neurology, Daping Hospital, Third Military Medical University, 10 Changjiang Branch Road, Yuzhong District, Chongqing 400042, China; 2 Department of Rehabilitation Physical Therapy, Southwest Hospital, Third Military Medical University, Gao tan yan street, Shapingba District, Chongqing 400038, China; 3 College of Biomedical Engineering, Chongqing University, Chongqing 400044, China; 4 Department of Development and Regeneration Key Laboratory of Sichuan Province, Department of Histo-embryology and Neurobiology, Chengdu Medical College, Chengdu 610083, PR China

**Keywords:** Toll-like receptor 4, MyD88, TRIF, Inflammation, Intracerebral hemorrhage, Heme

## Abstract

**Background:**

Inflammatory injury plays a critical role in intracerebral hemorrhage (ICH)-induced neurological deficits; however, the signaling pathways are not apparent by which the upstream cellular events trigger innate immune and inflammatory responses that contribute to neurological impairments. Toll-like receptor 4 (TLR4) plays a role in inflammatory damage caused by brain disorders.

**Methods:**

In this study, we investigate the role of TLR4 signaling in ICH-induced inflammation. In the ICH model, a significant upregulation of TLR4 expression in reactive microglia has been demonstrated using real-time RT-PCR. Activation of microglia was detected by immunohistochemistry, cytokines were measured by ELISA, MyD88, TRIF and NF-κB were measured by Western blot and EMSA, animal behavior was evaluated by animal behavioristics.

**Results:**

Compared to WT mice, TLR4^−/− ^mice had restrained ICH-induced brain damage showing in reduced cerebral edema and lower neurological deficit scores. Quantification of cytokines including IL-6, TNF-α and IL-1β and assessment of macrophage infiltration in perihematoma tissues from TLR4^−/−^, MyD88^−/− ^and TRIF^−/− ^mice showed attenuated inflammatory damage after ICH. TLR4^−/− ^mice also exhibited reduced MyD88 and TRIF expression which was accompanied by decreased NF-κB activity. This suggests that after ICH both MyD88 and TRIF pathways might be involved in TLR4-mediated inflammatory injury possibly via NF-κB activation. Exogenous hemin administration significantly increased TLR4 expression and microglial activation in cultures and also exacerbated brain injury in WT mice but not in TLR4^−/− ^mice. Anti-TLR4 antibody administration suppressed hemin-induced microglial activation in cultures and in the mice model of ICH.

**Conclusions:**

Our findings suggest that heme potentiates microglial activation *via *TLR4, in turn inducing NF-κB activation *via *the MyD88/TRIF signaling pathway, and ultimately increasing cytokine expression and inflammatory injury in ICH. Targeting TLR4 signaling may be a promising therapeutic strategy for ICH.

## Background

Intracerebral hemorrhage (ICH) is a common and serious acute cerebrovascular disease (CVD), accounting for approximately 10-15% of CVD cases. Incidence, fatality and disabling rate are high for ICH, and most of the survivors suffer from apparent disability. Although there is a significant body of research on the events following ICH, the signaling pathways by which the initial cellular events trigger innate immune and inflammatory responses that contribute to neurological impairments are not apparent [1-3]. Following ICH, a progressive inflammatory process arises from perihematoma edema [4-7], where blood infiltrating the brain parenchyma leads to disruption of the blood-brain barrier (BBB) [8], infiltration of neutrophil and macrophage [9], hematoma expansion [10] and ultimately neuronal death.

Innate immunity and inflammatory responses may contribute to neurological deficits possibly through release of endogenous ligands, which exert functions largely through Toll-like receptors (TLRs) [11,12]. TLRs were the first and well characterized pattern-recognition receptors (PRRs) to be identified. To date, 10 and 13, respectively, functional TLRs which recognize distinct PAMPs derived from viruses, bacteria, mycobacteria, fungi and parasites have been identified in humans and in mice, respectively [13,14]. Upon recognition of respective PAMPs, TLRs recruit a specific set of adaptor molecules that harbor the TIR domain, such as MyD88 and TRIF, and initiate downstream signaling events including nuclear factor kappa B (NF-κB) that leads to the expression of gene encoding inflammation-associated molecules and cytokines [15]. TLR4 interacts with the adapter protein myeloid differentiation factor 88 (MyD88) or/and TIR-domain-containing adapter-inducing interferon-β (TRIF) to activate NF-κB, which regulates the gene expression of inflammatory mediators such as the cytokines interleukin (IL)-1α and-1β, tumor necrosis factor (TNF)-α, and IL-6 [16,17]. Recently, it has been demonstrated that TLR4 contributes to inflammatory injury in central nervous system infection and cerebral ischemia [18,19]. TLR4 is an important contributor to microglial activation and known to initiate an inflammatory cascade in response to various brain injuries. However, the role of TLR4 in ICH-induced inflammatory injury remains unclear.

In the hemorrhage brain, erythrocyte lysis occurs following an intracerebral bleed [6,10], releasing free heme (iron protoporphyrin IX), which is degraded by heme oxygenase into ferrous iron, carbon monoxide, and biliverdin. Iron overload and hemoglobin toxicity has been thought to contribute to microglia activation, brain edema, oxidative stress, and upregulation of cytokine expression [4,5,7]. A recent report suggests that heme activates TLR4 to induce TNF-α secretion [20]. Also, after ICH, upregulation of TLR4 and activation of NF-κB in perihematoma tissues have been reported [21,22]. However, the exact mechanism of the TLR4 signaling pathway in ICH is not fully understood. Therefore, we propose a hypothesis that following ICH, hematoma components (i.e. heme) may act on TLR4 expressed on inflammatory cells, and activate gene transcription through the TLR4 downstream signaling pathway, which results in the production of inflammatory factors and ultimately leads to inflammatory injury and neurological deficits. To test this hypothesis, we investigated in this study the role of TLR4 in ICH-induced inflammatory injury and explore the endogenous trigger and possible signaling pathway involved in TLR4-mediated inflammatory response following ICH.

## Materials and methods

### Animals

C57BL/6 mice (male, 8-10 weeks, 20-24 g) were obtained from the Animal Center of the Third Military Medical University (Chongqing, China). Transgenic line TLR4^−/−^, MyD88^−/− ^and TRIF^−/− ^mice (8-10 weeks, 21-22 g) were purchased from American Jackson Laboratories (Bar Harbor, ME, USA) and were backcrossed to a C57BL/6 more than 8 times. Animals were housed in individual cages with free access to sterile acidified water and irradiated food in a specific pathogen-free facility at the Third Military Medical University. Experiments were conducted in accordance with animal care guidelines approved by the Animal Ethics Committee of the Third Military Medical University.

### Mouse model of intracerebral hemorrhage (ICH)

A modified mouse model of ICH was used [23]. Mice were anesthetized with intraperitoneal chloral hydrate (40 mg/kg) and placed in a stereotaxic frame (Stoelting, Kiel, WI, USA). A micro-sample instrument was lowered into the center of the striatum by craniotomy under stereotactic guidance at the following coordinates relative to bregma: 1 mm anterior, 2.5 mm lateral, and 4 mm deep. A volume of 25 μL of autologous whole blood was infused at 2.5 μL/min over a period of 10 min. The needle was held in place for another 10 min after the infusion to prevent leakage. Craniotomy was then sealed with bone wax, and the scalp was closed with suture. Control mice were infused with 25 μL of 0.9% saline. We maintained the room temperature at about 25°C during and after surgery, and exposed the animals to incandescent lighting to keep their rectal temperature at 37 ± 1°C until palinesthesia. An arterial catheter to measure arterial blood pressure (MABP) was placed in the femoral artery. Physiological parameters such as MABP (mm Hg), heart rate (per minute), glucose (mg/dL), arterial pH, arterial pO_2_, base excess, Hb (g/L), Laser Doppler, and body weight (g) were measured 30 minutes before and after ICH modeling. Treatment of ICH did not affect these parameters to a significant extent. Different experimental groups were included: WT sham (*n *= 69), WT ICH (*n *= 51), TLR4^−/− ^sham (*n *= 15), TLR4^−/− ^ICH (*n *= 39), MyD88^−/− ^sham (*n *= 6), MyD88^−/− ^ICH (*n *= 12), TRIF^−/− ^sham (*n *= 6), TRIF^−/− ^ICH (*n *= 12). Some mice were used for several experiments.

In order to investigate the effect of TLR4 depletion on heme-induced inflammation, 25 μL of hemin (Lundbeck Inc., Deerfield, IL 60015, USA, reconstituted with sterile water to a concentration of approximately 7 mg/mL. NDC numbers: 67386-701-54. Lot No: 14674-Z7.) was injected into the striatum in mice as described previously [24]. The mice were subjected to experiments 24 h later. The doses The used in this experiment were determined according to the literature [24] and according to our *ex vivo *study in which these doses led to significant cerebral edema and neurological deficits in mice. In the control group, 25 μL of vehicle (sterile buffer of 5 mg/ml sodium carbonate and 6.98 mg/ml of sorbitol) was injected.

### Real-time reverse transcription polymerase chain reaction (real-time RT PCR)

Total RNA was extracted from perihematoma tissues, about 4 × 4 × 4 mm^3 ^volume (blood clot as core under a stereomicroscope) on day 1, 2, 3, and 5 post-ICH according to previous methods [25] and from microglial cultures using Trizol reagent (Invitrogen, Gaithersburg, MD, USA) according to the manufacturer's instructions. cDNA was synthesized using the iScript cDNA Synthesis Kit (Bio-Rad) and the real-time RT-PCR was carried out on a Biorad I-Cycler with the IQ^TM ^SYBER^® ^Green Supermix (Bio-Rad) in 96-well plates. Primers were purchased from Shanghai Sangon Biological Engineering (Shanghai, China). The primer sequences were as follows: TLR4, forward: 5'-GTCAGTGTGATTGTGGTATCC-3', reverse: 5'-ACCCAGTCC-TCATTCTGACTC-3'. To quantify measurements of gene expression, a threshold cycle value (C_T_) was calculated, using the Δ ΔC_T _method as previously described [26].

### Immunohistochemical staining

On day 1, 2, 3, and 5 post-ICH, mice were anesthetized and intracardially perfused with phosphate buffered saline (PBS), followed by 4% paraformaldehyde (PFA) as described previously [27,28]. Brains were removed and post-fixed in 4% PFA at 4°C overnight. Thirty micro brain sections were obtained with microtome. Double-fluorescent immunohistochemistry was performed to identify TLR4 expression in tissue according the conventional immunostaining method. Primary antibodies used in this experiment included: goat anti-mouse TLR4 (1:200, Abcam, Cambridge, UK), rabbit anti-mouse βIII-tubulin (1:1000, Abcam Cambridge, UK), rat anti-mouse CD11b (1:200, eBioscience San Diego, CA, USA) and rabbit anti-mouse GFAP (1:1000, Abcam Cambridge, UK). Secondary antibodies included Alexa Flour^® ^488 (goat anti-rat IgG (H + L), goat anti-rabbit IgG (H + L) and Alexa Flour^® ^546 (donkey anti-goat IgG (H + L); Invitrogen, Carlsbad, CA, USA). Quantification of immunohistochemistry was performed as previously described, the results were shown as "% area of perihematoma tissue region positive" [29]. TLR4-positive cells were accessed blindly and a scale of 0, 1, 2, 3, 4 and 5, respectively, indicates the area-percent with an expression of 0%, ≤10%, 11-25%, 26-45%, 46-75%, and >76%, respectively.

To assess macrophage infiltration, brain sections were incubated with rabbit anti-mouse CD68 (1:200, Abcam, Cambridge, UK) at 4°C overnight and the anti-rabbit HRP/DAB detection system was used to visualize the expression. Three sections were prepared for each brain tissue specimen obtained from the perihematoma region and CD68-positive macrophages were counted within 20 consecutive high-power fields (×400) as previously described [29]. Cell counting was performed by two independent observers in a blind manner. Images were acquired using a digital confocal microscope (Leica TCS Sp5, Mannheim, Germany) and Nikon optical microscope (Nikon Eclipse 90i, Tokyo, Japan). IPP 6. 0 image processing software was used to count the number of CD68-positive cells. Image analysts were blind to all the experiments.

### Neurological deficit scores

After 3 day post ICH modeling, the mice were in stable condition, the neurological deficit tests were performed by behavioral measurement, including postural flexing test, circling or sidewalk, forelimb placing and foot fault test and repeated 3 times according to the method by Zhao X *et al. *[30].

### Measurement of cerebral water content of mice

To measure cerebral water content after ICH, mice were randomly selected from each group and euthanized. We levered the skull within 1 minute to take out brain tissues, blotted up the water on the surface of the left hemisphere with filter paper, and took the humid weights (GW) on an electronic balance. We then dried them for 24-48 h at 95°-100°C in an Electro-Thermostatic Blast Oven and took their dry weights (DW). Cerebral water contents were calculated by the formula: cerebral water content% = (GW - DW)/GW × 100%.

### Enzyme-linked immunosorbent assay (ELISA)

ELISA was performed as per the manufacturer's instructions (Dakewe Biotech, Shenzhen, China) to assess the concentrations of TNF-α, IL-6, and IL-1β in brain tissues obtained from the perihematoma region. Brain tissues (80 mg) were centrifuged at 12000 g and the supernatant was collected for analysis. The detection threshold of this assay was <1 pg/mL.

### Western blot analysis

Western blot analysis was performed as previously described [31]. Briefly, proteins were separated from perihematoma tissues (80 mg) or microglia (1 × 10^5^) by SDS polyacrylamide gel electrophoresis and transferred onto polyvinylidene fluoride (PVDF) membranes (Amersham Pharmacia). The PVDF membranes were incubated with the primary antibodies, including: rabbit anti-mouse MyD88 (for perihematoma tissues, 1:500, Abcam, Cambridge, UK), rabbit anti-mouse TRIF (for perihematoma tissues, 1:500, Abcam, Cambridge, UK) and rabbit anti-mouse TLR4 (for microglia, 1:500, Abcam, Cambridge, UK), followed by incubation with peroxidase-conjugated secondary antibodies (1:2000, Jingmei, China). The signals were detected with an ECL system (Amersham Pharmacia). The same membranes were probed with antibody for glyceradehyde-3-phosphate dehydrogenase (GAPDH) after being washed with stripping buffer. The signals were quantified by scanning densitometry and computer-assisted image analysis.

### Determination of NF-κB activity by electrophoretic mobility shift assay

Electrophoretic mobility shift assay was performed as previously described [31,32]. Nucleoprotein was extracted from brain tissue according to the manufacturer's instructions. Ten microgram of nucleoprotein of each sample was incubated with the reaction buffer at room temperature for 15 min, then P^32^-labeled oligonucleotide (5'-GGGGACTTTCC-3'; Life Technologies, Gaithersburg, MD, USA), which binds to NF-κB, was added to the reaction buffer and incubated for 15 min. After incubation for 20 min at 25°C, the reaction mixture was subjected to 6% non-denaturing polyacrylamide gel electrophoresis. Autoradiography was performed at room temperature. Finally, the images were analyzed using an Bio-Rad Image Analyzer (CA, USA) and the results were expressed as optical density (OD).

### Microglia culture and treatment

Primary microglia culture was performed as previously described [33]. Cerebral hemispheres of 1-day old postnatal mice (both wild type and TLR4^−/− ^mice) were digested with 0.1% trypsin. The cells were seeded into a six-well plate coated with poly-L-lysine and fed with Dulbecco's Modified Eagle Media (DMEM; Sigma, St. Louis, MO, USA) containing 10% fetal bovine serum (FBS; Hyclone, Logan, UT/USA). Culture media were refreshed twice per week for 2 weeks. Microglia were detached by gentle shaking and filtered through a nylon mesh to remove astrocytes. After centrifugation at 1000 × g for 10 min, the cells were resuspended in fresh DMEM supplemented with 10% FBS and plated at a final density of 5 × 10^5 ^cells/mL on a poly-L-lysine-coated 6-well culture plate. The following day, cells were subjected to the experiments. The cell purity was determined by immunohistochemical staining using microglia specific antibody CD11b. The microglia cultures used were >95% pure.

To assess the TLR4 expression after stimulation, WT microglia were stimulated with 30 μM each of hemin, bilirubin (Sigma, Shanghai, China), Fe^2+ ^(FeSO_4_, Sigma, Shanghai, China) for 24 h. Western blot was used to assess TLR4 expression. To determine the activation of microglia after stimulation, 30 μM each of hemin, bilirubin, FeSO_4_, LPS((100 ng/mL), hemin + polymyxin B (PB, New Bedford Laboratorie, MA, USA; 20 μg/mL) and LPS + PB for 24 h, TNF-α release was determined by ELISA.

To further evaluate microglial activation in response to stimulus, microglial cultures from WT and TLR4^−/− ^were stimulated for 24 h with 1) vehicle, 2) 30 μM of hemin. WT microglial cells were also stimulated with combinations of 3) Hemin + 10 μg/mL anti-TLR4 monoclonal unconjugated antibody Mts510 (LifeSpan BioSciences, Seattle, WA, USA), 4) Hemin + IgG. The dose of the anti-TLR4 antibody was determined as previously described [32]. The release of TNF-α, IL-1β and IL-6 was analyzed by ELISA.

### Anti-TLR4 monoclonal antibody

We used anti-mouse TLR4 monoclonal antibody to neutralize TLR4 in mice. Mice were divided into four groups (n = 6): 1) WT mice injected with saline, 2) TLR4^−/− ^mice, 3) WT mice injected with anti-TLR4 antibody, and 4) WT mice injected with control antibody IgG2α. For antibody administration, 200 μg of anti-mouse TLR4 monoclonal unconjugated antibody Mts510 (LifeSpan BioSciences, Seattle, WA, USA) were injected i.v. immediately after ICH. Mice in group 4) received class-match control rat anti-mouse mAb (IgG2α, 200 μg/mL, LifeSpan Biosciences, Seattle, WA, USA). The dose of antibodies used was based on our previous findings [34]. Cerebral water content and extent of neurological impairment were measured 3 days after ICH. Meanwhile, ELISA was used to assess expression of TNF-α, IL-1β and IL-6 in damaged tissue. Macrophage infiltration was evaluated with CD68 immunohistochemistry.

### Statistical analysis

Results were shown as mean ± standard deviation (SD). Multiple groups were compared using one-way or two-way analysis of variance and Student-Newman-Keuls test in post hoc tests. Statistical Package for the Social Sciences (SPSS) 11.5 software (Chicago, IL, USA) was used for statistical analyses. A probability value (*p*) < 0.05 was deemed statistically significant.

## Results

### ICH-induced TLR4 upregulation in perihematoma brain tissues

**Figure 1:**
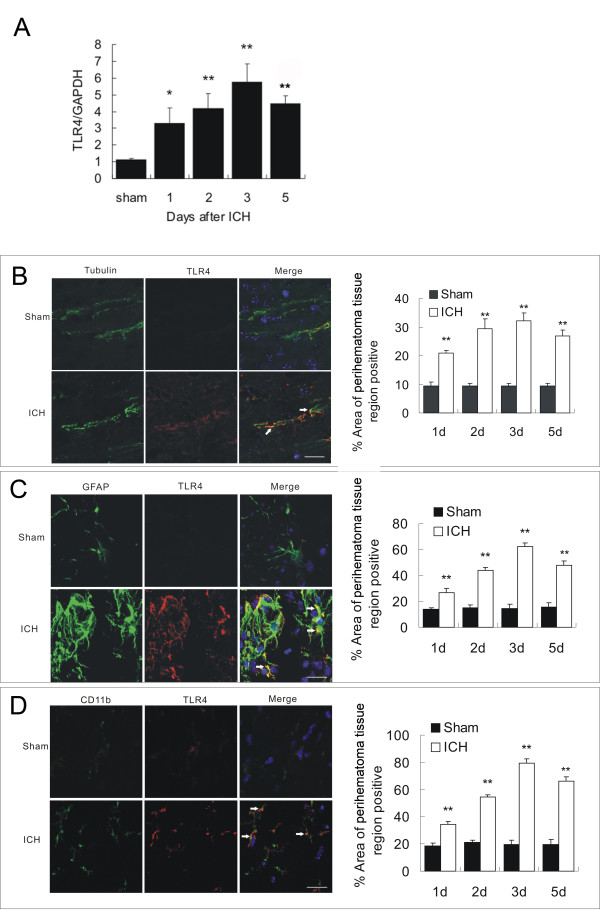
**TLR4 mRNA and protein expression after ICH**. **A**) Real-time RT-PCR shows an upregulation of TLR4 mRNA expression in perihematoma tissues in WT mice (n = 6) on days 1, 2, 3, and 5 post ICH. **B**) TLR4 co-labeling with tubulin-positive neuron (arrows, n = 6). **C**) TLR4 co-labeling with GFAP-positive astrocytes (arrows, n = 6). D) TLR4 co-labeling with CD11b-positive microglia. Compared to sham control mice (n = 6), ICH induced significant increase in TLR4 protein. *P < 0.05, **P < 0.01 *vs*. sham control. Values (mean ± SD) are representative of two independent experiments. Bar = 20 μM.

To explore the role of TLR4 in inflammation after ICH, we first analyzed TLR4 mRNA expression on day 1, 2, 3, and 5 post-ICH using a real-time RT-PCR. The result showed that TLR4 mRNA was significantly upregulated in perihematoma brain tissues following ICH at all tested time-points when compared to sham-operated control mice (Figure 1A). TLR4 mRNA expression peaked on day 3 (*P *< 0. 01) and started decreasing on day 5 (*P *0. 01). We next performed double-immunofluorescence staining to assess cellular expression of TLR4. Anti-tubilin, anti-GFAP, and anti-CD11b, respectively, were used as cell marker for neuron (Figure 1B), astrocyte (Figure 1C) and microglial cell (Figure 1D), respectively. We found that TLR4 expressed in all these cell types and the protein expression was significantly upregulated when compared to that of the sham group (*P *< 0.01; Figure 1B-D. Consistent with the TLR4 mRNA upregulation, TLR4 expression also peaked on day 3 and began decreasing on day 5 following ICH. Although both neuron and reactive astrocyte exhibited TLR4 expression, double-fluorescent staining showed that TLR4 predominantly expressed in CD11b-positive cells.

### TLR4^−/− ^mice showed significantly lower levels of inflammation and neurological impairment following ICH

**Figure 2:**
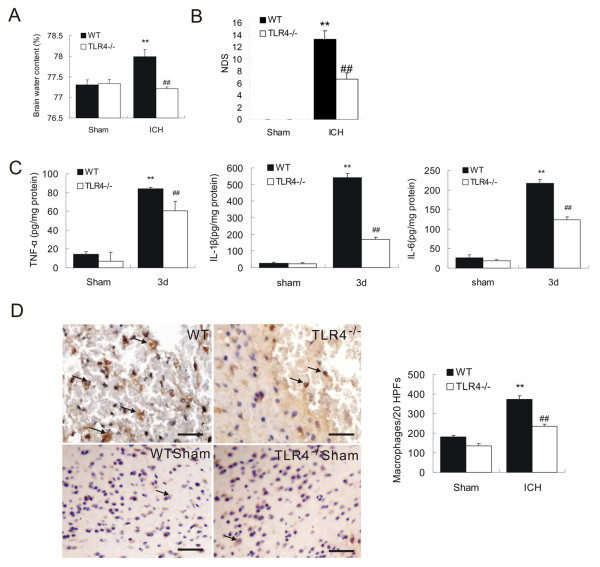
**TLR4^−/− ^mice displayed attenuated brain injury and decreased neuroinflammation**. TLR4^−/− ^had decreased brain water content (**A**, n = 3) and decreased NDS (**B**, n = 3). ELISA showed that TLR4^−/− ^mice had a marked decrease in release of IL-6, TNF-α, and IL-1β (**C**, n = 3) on day 3 post-ICH. D) Immunohistochemistry of CD68 showed decreased macrophage infiltration in TLR4^−/− ^mice on day 3 post-ICH (n = 3). **P < 0.01 *vs*. sham group; ^##^*P *< 0. 01 *vs*. WT group; Bar = 50 μM in D. Values (mean ± SD) are representative of three independent experiments.

To further assess the role of TLR4 in the inflammatory cascade in response to ICH, we employed TLR4 knockout mice (TLR4^−/−^). We induced ICH in TLR4^−/− ^mice and then evaluated brain damage and neurological impairments 3 days after ICH when we observed the maximum upregulation of TLR4 in WT mice with ICH. Compared to WT mice, TLR4^−/− ^mice showed a significantly lower brain water content (*P *< 0.01, *n *= 3, Figure 2A) and neurological deficit scores (NDS) (*P *< 0.01, *n *= 3, Figure 2B). These suggested that TLR4^−/− ^mice had less extent of neurological impairments after ICH. Next, we detected inflammatory cytokines expression in TLR4^−/− ^mice after ICH. ELISA showed that TLR4^−/− ^mice exhibited significantly lower (*P *< 0.01, *n *= 3) expression of IL-6, TNF-α, and IL-1β in perihematoma tissues 3 days after ICH (Figure 2C). Finally, we examined macrophage infiltration in perihematoma tissues and found that the extent of macrophage infiltration is dramatically decreased (*P *< 0.01, *n *= 3, Figure 2D) as shown in decreased CD68-positive cells (arrows in Figure 2D) in 20 consecutive high-power fields (20 HPFs) obtained from the perihematoma region.

### TLR4-mediated inflammation after ICH *via *MyD88/TRIF signaling pathway and NF-κB activation

It has been established that TLR4 exerts its functions through intracytoplasmic TIR domain-containing adaptors, such as MyD88 and TRIF. MyD88, a common TIR domain-containing adaptor, is essential for the induction of inflammatory cytokines triggered by TLR4 [16,17]. TRIF is implicated in the TLR4-mediated MyD88-independent pathway. Both MyD88 and TRIF modulate TLR4-mediated activation of immunity and inflammation. To investigate which signal pathway is involved in TLR4-mediated inflammatory response after ICH, we employed MyD88^−/− ^and TRIF^−/− ^mice.

**Figure 3:**
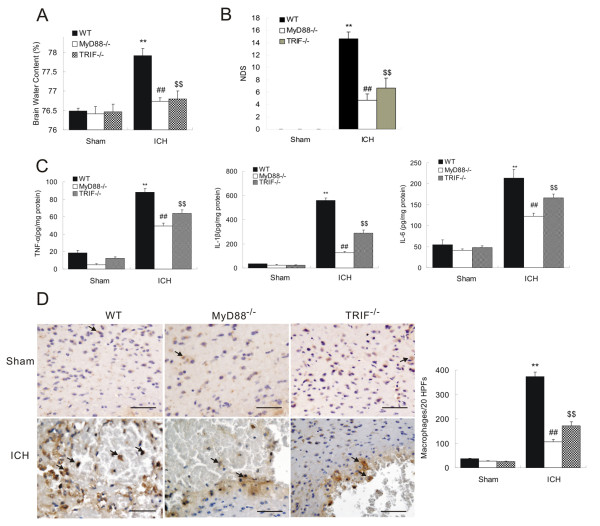
**Declined neurologic deficits and inflammation in MyD88 ^−/− ^and TRIF^−/− ^mice**. Compared to WT mice, both MyD88^−/− ^and TRIF^−/− ^mice had decreased brain water content (**A**), NDS (**B**), (**C**) Both MyD88^−/− ^and TRIF^−/− ^mice showed decreased protein levels of IL-6, TNF-α, and IL-1β, and (**D**) immunohistochemistry of CD68 showed decreased macrophage infiltration in the perihematoma region of MyD88^−/− ^and TRIF^−/− ^brain on day3 post-ICH. **P < 0.01 *vs*. sham group; ^##^*P *< 0. 01 *vs*. WT group. ^$$^*P *< 0.01 *vs*. WT group, Bar = 50 μM in D. Values (mean ± SD, n = 3 for each group) are representative of three independent experiments.

First, we evaluated the extent of ICH-induced brain damage and inflammatory responses in MyD88^−/− ^and TRIF^−/− ^mice. We found that compared to WT mice, both MyD88^−/− ^and TRIF^−/− ^mice had significantly less impairments after ICH, indicated by decreased cerebral water content (Figure 3A) and lower neurological deficit score (Figure 3B). Furthermore, we quantified inflammatory cytokine expression and macrophage infiltration in perihematoma tissues of these mice. ELISA showed that 3 days after ICH WT mice displayed dramatic increase in TNF-α, IL-1β, and IL-6 levels compared to the sham group. On the contrary, both MyD88^−/− ^and TRIF^−/− ^exhibited significant lower expression of these cytokines compared to WT mice, although significantly higher than the sham control (Figure 3C). This suggested that depletion of either MyD88 or TRIF dramatically suppressed ICH-induced cytokine expression. Reduced inflammation in these mice was also supported by the observation that MyD88^−/− ^and TRIF^−/− ^had significantly lower macrophage infiltration compared to WT mice, as shown in reduced CD68-positive cells (*P *< 0.01) in perihematoma tissues (Figure 3D). Taken together, these findings indicate that both MyD88 and TRIF signaling pathway might be involved in the TLR4-mediated inflammatory process following ICH.

**Figure 4:**
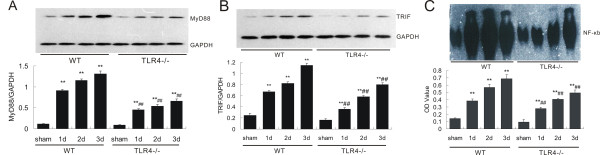
**TLR4^−/− ^mice showed reduced MyD88 and TRIF protein level and NF-κB activity**. Western blot showed TLR4^−/− ^had decreased expression of MyD88 (**A**) and TRIF (**B**). Electrophoretic mobility shift assay showed decreased NF-κB activity in TLR4^−/− ^(**C**) **P < 0.01 *vs*. sham group, ^##^*P *< 0. 01 *vs*. WT group. Values (mean ± SD, n = 3 for each group) are representative of three independent experiments.

To ask whether MyD88 or TRIF is the downstream pathway in TLR4-mediated inflammation following ICH, we assessed MyD88 and TRIF protein expression in perihematoma tissues from TLR4^−/− ^and WT mice. Western blot results showed that WT mice showed a significant increase in MyD88 and TRIF protein expression in response to ICH injury, while TLR4^−/− ^mice had much lower MyD88 and TRIF expression compared to WT mice at all tested time-points (*P *< 0.01, Figure 4A and 4B). This suggests that ICH-induced TLR4 activation triggered both MyD88 and TRIF expression, while abolishing TLR4-suppressed ICH-induced MyD88 and TRIF upregulation.

Activation of transcription factor NF-κB is a downstream event of the TLR4/MyD88 signaling pathway [16,17]. NF-κB plays a critical role in the inflammatory response by translocating into the nucleus and regulating the transcription of inflammatory genes [35]. We therefore compared NF-κB activity in TLR4^−/− ^and WT mice following ICH using an electrophoretic mobility shift assay, and found that ICH-induced increase of NF-κB activity in TLR4^−/− ^mice was significantly lower than the increase in WT mice at all tested time-points (*P *< 0. 01; Figure 4C). Overall, our results suggest that MyD88 and TRIF might both participate in TLR4-mediated inflammation in response to ICH *via *activation of NF-κB and modulating expression of inflammatory cytokines.

### Heme potentiated microglia activation and induced TLR4-mediated inflammatory injury

**Figure 5:**
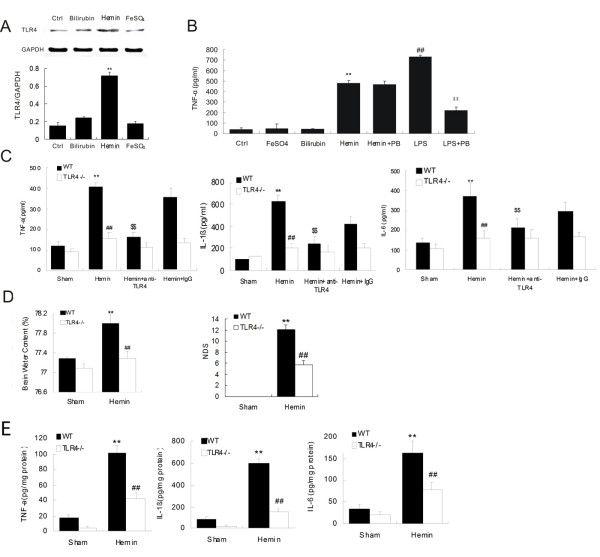
**Heme stimulates microglia activation and induces brain inflammatory injury *via *TLR4**. (**A**) Stimulation with bilirubin, Hemin and FeSO_4 _influenced TLR4 expression on cultured microglial cells. **P < 0.01 *vs*. control group (*n *= 6) (**B**) TNF-α release in microglial cells in response to stimulus. **, ^##^*P *< 0.01 *vs*. control group, ^$$^*P *< 0.01 *vs*. LPS group (*n *= 6). (**C**) TNF-α, IL-1β, and IL-6 expression in microglial cells in response to stimulus. **P < 0.01 *vs*. control group, ^##, $$^*P *< 0.01 *vs*. WT group, n = 6. Hemin treatment decreased brain water content (**D**) and NDS (D) in TLR4^−/− ^mice. **P < 0.01 *vs*. control group, ^##^*P *< 0.01 *vs*. WT group *n *= 6. (**E**) Hemin treatment increased cytokines release in TLR4^−/− ^mice. **P < 0.01 *vs*. control group, ^##^*P *< 0.01 *vs*. WT group n = 6. Values (mean ± SD) are representative of two independent experiments.

Heme and iron metabolism are of considerable importance in neuropathogenesis following traumatic injury and hemorrhagic stroke [36]. Free heme is released through hemoglobin lysis following an intracerebral bleed, and then degraded by heme oxygenase to form free iron [7]. The above data showed that TLR4 was significantly upregulated in microglia following ICH, however, the event that triggered the activation of TLR4 remained unclear. Free heme and iron are potent inducers of inflammation, and are highly toxic to brain tissues [6,10]. To test the hypothesis that heme and iron trigger activation of TLR4 after ICH, we investigated the effect of exogenous hemin and iron on the expression of TLR4 and cytokine release in both *in vitro *and *in vivo *< models. First, using primary cultured microglial cells, we found that hemin treatment significantly increased TLR4 expression (*P *< 0. 01) in cultured microglia while bilirubin and FeSO_4 _showed no effect (Figure 5A). Moreover, the expression of TNF-α by activated microglia was significantly increased (*P *< 0.01). A major concern when characterizing any putative ligand of TLR4, especially in an endogenous environment, is the possible presence of microbial derived contaminants. Determination of the content of endotoxin by the limulus assay demonstrated that hemin preparations used in the study were free of any detectable LPS. Furthermore, hemin induced TNF-α in the presence of polymyxin B in a concentration that fully abolished the LPS effect. Hemin also induced the secretion of TNF-α by microliga in the presence of polymyxin B (Figure 5B).

Next, we investigated the effects of hemin stimulation on microglia culture derived from TLR4^−/− ^mice. We observed that administration of hemin significantly induced the expression of TNF-α, IL-1β, and IL-6 (Figure 5C) in microglia from WT mice but not from TLR4^−/− ^mice, suggesting that TLR4 depletion suppressed hemin-stimulated microglial activation. Co-administration of hemin with anti-TLR4 antibody to WT microglial cells also showed decreased expression of TNF-α, IL-1β, and IL-6 (Figure 5C). Co-administration of IgG as a control for anti-TLR4 antibody showed no effect. These data suggest that heme might potentiate microglial activation *P *< TLR4 and both genetic depletion and antibody neutralization of TLR4 in microglial cells lead to suppression in heme-induced microglial activation.

To test the hypothesis that heme causes TLR4-mediated inflammatory injury after ICH, we further examined the effect of exogenous hemin application in the mouse model. Hemin was stereotactically injected into the striatum in WT and TLR4^−/− ^mice. One day after injection, we evaluated the extent of brain injury and inflammation as described above. Our data showed that administration of hemin dramatically induced brain damage in WT mice, as demonstrated in significant increase in brain water content (*P *< 0.01, *n *= 6, Figure 5D) and NDS (*P *< 0.01, *n *= 6, Figure 5D) of WT mice. However, the deteriorating effect of hemin was not found in TLR4^−/− ^mice as no obvious change was detected in brain water content and NDS. Meanwhile, IL-6, TNF-α, and IL-1β expression increased significantly in perihematoma tissues of WT mice after hemin injection (Figure 5E) but not in TLR4^−/− ^mice, suggesting that depletion of TLR4 suppressed the hemin-induced inflammatory process after ICH. Together with *in vitro *data, these findings suggest that heme might trigger TLR4-mediated inflammatory injury following ICH possibly through activating microglial cell and inflammatory response, and depletion of TLR4 could protect ICH brain from heme-induced-brain damage.

### TLR4 monoclonal antibody conferred significant neuroprotection following ICH

**Figure 6:**
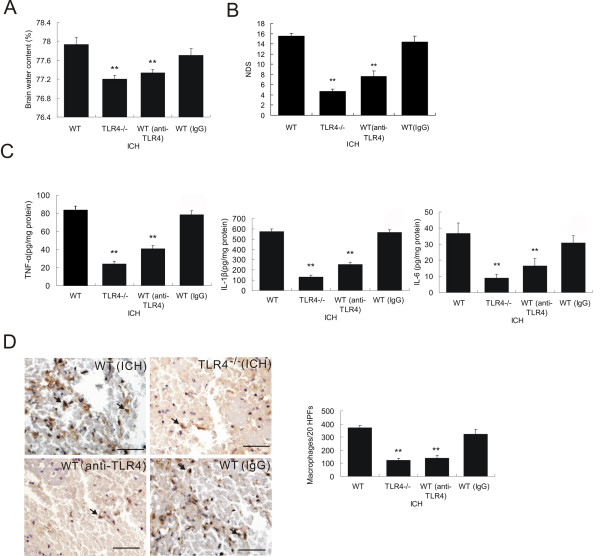
**Administration of anti-TLR4 monoclonal antibody immediately after ICH provided neuroprotection**. Administration of antibody against TLR4 in WT mice of ICH decreased brain water content (A) and NDS (B). Administration of antibody against TLR4 in WT mice significantly decreased cytokine release (C) and macrophage infiltration (D) in response to ICH. **P < 0.01 *vs*. WT group. Scale bar = 50 μM (D). Values (mean ± SD, n = 6 for each group) are representative of three independent experiments.

The above experiments showed a beneficial effect of TLR4 depletion in ICH-induced brain damage. In our previous study, we also showed that intravenous administration of anti-TLR4 antibody 30 min prior to occlusion of the middle cerebral artery attenuated ischemia-reperfusion damage and improved mice behavioral performance [34]. In this study, we were interested in assessing the effect of neutralizing TLR4 using Mts510 in mice with ICH. We found that mice that received Mts510 showed significantly lower cerebral water content (Figure 6A) and neurological impairments (Figure 6B) compared to WT mice and WT mice with IgG control. This antibody-mediated neuroprotection effect was similar to the observation in TLR4^−/− ^mice. Antibody administration prior to ICH also showed inhibition of cytokine expression (Figure 6C) and macrophage infiltration (Figure 6D), indicating a reduced inflammatory process in TLR4-depleted mice. These results indicate that administration of anti-TLR4 monoclonal antibody prior to ICH exhibits significant neuroprotection, which possibly acts through suppression of cytokine expression and macrophage infiltration in the damaged region of the ICH brain.

## Discussion

**Figure 7:**
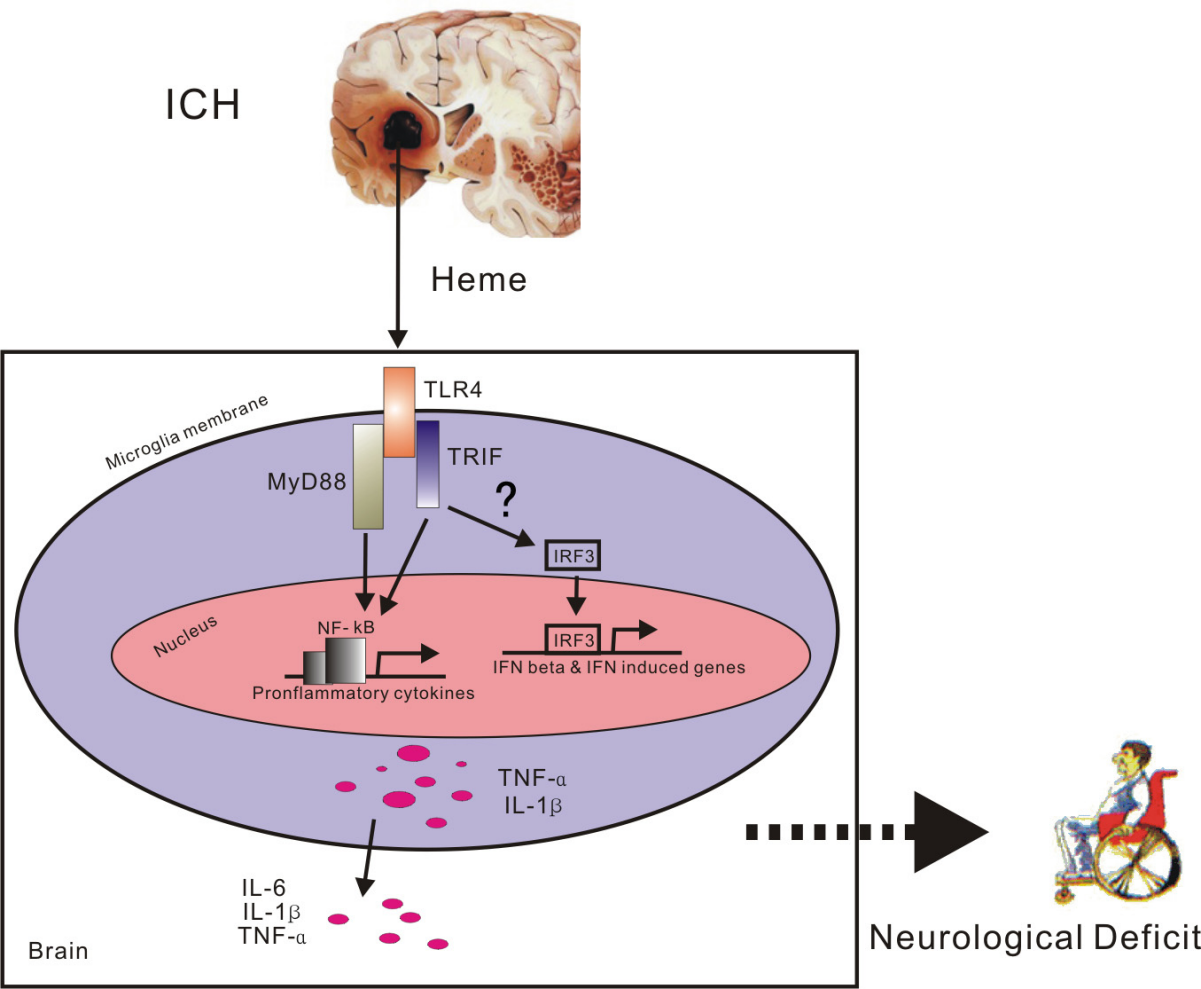
**Schematic diagram of the Heme/TLR4/MyD88 and/or TRIF hypothesis in ICH**. Heme activates TLR4-mediated inflammatory injury *via *the MyD88/TRIF signaling pathway in intracerebral hemorrhage mouse brain.

Inflammatory injury plays an important role in ICH-induced neurological deficit. However, the signaling pathways by which the upstream cellular events trigger innate immune and inflammatory responses that contribute to neurological impairments are not apparent. Teng *et al. *[21] reported that TLR4 and NF-κB were unregulated in perihematoma tissues, suggesting that TLR4 signaling was involved in ICH-induced brain injury. In the present study, we explored the role of TLR4 in ICH-induced inflammatory injury and tested the hypothesis that heme and iron, products of erythrocyte lysis, could trigger TLR4-mediated inflammatory damage *via *activation of NF-κB during ICH. We found that ICH significantly upregulates TLR4 expression in microglial cells and induces NF-κB activity possibly *via *the MyD88/TRIF signaling pathway, which modulates cytokine expression and macrophage infiltration and results in inflammatory damage (Figure 7). We further showed that after ICH heme triggers TLR4-mediated inflammatory injury. Neutralizing TLR4 with antibody at the time of ICH provides beneficial neuroprotection. To our knowledge, the present study demonstrates, for the first time, that heme induces TLR4 activation which might be an upstream event in the inflammatory process in response to ICH, suggesting that TLR4 is a promising target for prevention and therapeutic treatment of ICH.

Previous studies indicated that TLR4 is expressed in many type cells, like macrophage, hepatocyte and renal tubular cells [29,37,38]. In CNS, TLR4 has been reported to be expressed in both microglia and astrocytes, and even in neurons [39-41]. In accordance with previous results, we found that TLR4 expression increased markedly in microglial cells, indicating its involvement in ICH-induced inflammation. Our data also showed expression of TLR4 in neurons and astrocytes in perihematoma tissue, suggesting TLR4 in neurons and astrocytes may also play a role in the disease. The possible role of TLR4 in neurons and astrocytes in neuroinflammation of ICH needs to be further explored.

The TLR signaling pathway arises from intracytoplasmic TIR domains, which contain adaptors such as MyD88 and TRIF. MyD88 is essential for the induction of inflammatory cytokines whereas TRIF is mainly implicated in the TLR3- and TLR4-mediated MyD88-independent pathway [42,43]. Both MyD88 and TRIF can modulate TLR4-mediated inflammation. The ICH model in transgenic mice showed that abolishing of MyD88 and TRIF, respectively, led to reduced neurological deficits and reduced cytokine release and macrophage infiltration, which were similar to what we observed in TLR4^−/− ^mice. A reduced expression of MyD88 and TRIF in TLR4−/− mice in response to ICH injury is direct evidence of the involvement of MyD88 and TRIF in the TLR4 signaling pathway after ICH. Moreover, concomitant decrease in NF-κB activity was observed with a decrease in MyD88 and TRIF expression. In summary, this suggests that both MyD88 and TRIF are involved in TLR4-mediated inflammation after ICH and deletion of TLR4 suppresses ICH-induced activation of its downstream signaling pathway which in WT mice might contribute to inflammation-related neurological deficits. Our previous study showed that only the MyD88 but not the TRIF pathway was involved in TLR4-mediated ischemia damage [31], suggesting that although TLR4 participates in both ischemia- and hemorrhage-induced brain injury, the underlying signaling pathway is different. The downstream pathway involved in TLR4 activation may depend on the type of brain injury and initiating factors.

Free heme is released during erythrocyte lysis, and then degraded by heme oxygenase to form iron [7,44]. Morphologically, red cells maintain their biconcave shape for several days following ICH (~2 days in rats) [45]. *In vitro*, red cell lysis occurs slowly with the release of oxyhemoglobin starting after 2 days [46]. Accumulation of heme and iron in the perihematoma region is characterized by neurotoxicity, and causes acute inflammation through amplifying leukocyte activation and upregulating the expression of adhesion molecules and cytokine [44,47]. TLR4 has been identified as capable of binding to heme, and inducing TNF-α secretion [21]. In light of our observation of TLR4-mediated inflammatory injury after ICH, we therefore propose that accumulation of heme in perhematorma region might trigger TLR4-mediated inflammation in the ICH context. We test this hypothesis using both cultured microglia and transgenic mice models. *In vitro *studies showed that hemin induced TLR4 overexpression in microglial cells, but iron did not. Moreover, the expression of TNF-α by activated microglia was significantly increased only by hemin. Meanwhile, our results also showed that hemin induced microglia to release inflammatory factors *via *TLR4. This suggests a direct correlation between TLR4 and hemin-induced microglial activation, which was further supported by the observation that application of TLR4 antibody suppressed hemin-induced microglial activation. Previously, Figueiredo *et al. *[21] reported that only hemin but not Fe^2+ ^could activate macrophages *via *TLR4 to produce TNF-α, which was consistent with our results. However, Figueiredo *et al. *report that the monoclonal antibody (Mts510) did not block hemin-induced TLR4 activation in macrophages, rather it only blocks LPS induction, which was not consistent with our results. The discrepancy could be due to the different reagent used in the experiments. Endotoxin (LPS) contamination in the reagent could possibly affect experimental results. In order to exam the possible presence of endotoxin in heme reagents, the endotoxin level was determined using a standard endotoxin-specific Limulus amebocyte lysate reagent. The level of endotoxin in heme was 0.01 endotoxin units (1 EU/mL), which ruled out any effect of endotoxin. Therefore, our experimental results are not due to endotoxin contamination. The reason for the discrepancy was unclear, thus, the mechanism of heme in neuroinflammation of ICH needs to be further explored.

We further used an *in vivo *model to investigate the effect of heme-caused brain inflammation in the context of ICH. We found that exogenous administration of hemin induced inflammation in WT mice, while it had no marked effect on TLR4^−/− ^mice. Taken together with culture study, our data suggest that heme causes microglia activation and induces NF-κB activation *via *the TLR4/MyD88 and TRIF signaling pathway, and then induces neuroinflammation in ICH.

Finally, administration of TLR4 antibody to disrupt TLR4 signaling following ICH resulted in a neuroprotective effect, confirming a central role of TLR4 in ICH-induced inflammatory injury and suggests that TLR4 could be a potential therapeutic target in prevention and treatment of ICH.

## Conclusions

In conclusion, our findings reveal that heme triggers TLR4-mediated inflammatory injury *via *the MyD88/TRIF signaling pathway in intracerebral hemorrhage in mouse brain. This study suggests a central role of TLR4 in ICH-induced brain damage and provides a potential therapeutic target in prevention and treatment of ICH.

## Abbreviations

BBB: Blood-brain barrier; ELISA: Enzyme-linked immunosorbent assay; GFAP: Glial fibrillary acidic protein; HRP/DAB: Horseradish peroxidase/3,3'-Diaminobenzidine; ICAM: Intercellular adhesion molecule; ICH: Intracerebral hemorrhage; MyD88: Myeloid differentiation primary response protein; NF-κB: Nuclear factor kappa B; PB: Polymlxin B; TLR: Toll-like receptor; TNF: Tumor necrosis factor; TRIF: TIR-domain-containing adapter-inducing interferon-β (TRIF); WT: Wild type

## Competing interests

The authors declare that they have no competing interests.

## Authors' contributions

This study was based on the original idea of QWY. SL, QY and QZ carried out the molecular biology and behavioral studies and drafted the manuscript. FLL and YZ carried out behavioral studies and molecular biology. JZW performed mRNA studies. BYS provided the morphological laboratory and equipments. QWY performed data analyses. QWY and SL were responsible for supervising entire experiments, data analyses and writing manuscript. All authors read and approved the final manuscript.
